# The long-term effects of planting and harvesting on secondary forest dynamics under climate change in northeastern China

**DOI:** 10.1038/srep18490

**Published:** 2016-01-04

**Authors:** Jing Yao, Xingyuan He, Hongshi He, Wei Chen, Limin Dai, Bernard J. Lewis, Lizhong Yu

**Affiliations:** 1 State Key Laboratory of Forest and Soil Ecology, Institute of Applied Ecology, Chinese Academy of Sciences, Shenyang 110164, China; 2 Qingyuan Forest CERN, Chinese Academy of Sciences, Shenyang 110016, China

## Abstract

Unlike the virgin forest in the Changbaishan Nature Reserve in northeastern China, little research on a landscape scale has been conducted on secondary forests in the region under conditions of a warming climate. This research was undertaken in the upper Hun River region where the vegetation is representative of the typical secondary forest of northeastern China. The spatially explicit forest landscape model LANDIS was utilized to simulate the responses of forest restoration dynamics to anthropogenic disturbance (planting and harvesting) and evaluate the difference of the restoration process under continuation of current climatic conditions and climate warming. The results showed that: (1) The interaction of planting and harvesting has organizational scale effects on the forest. The combination of planting and harvesting policies has significant effects on the overall forest but not on individual species. (2) The area expansion of the historically dominant species *Pinus koraiensis* is less under climate warming than under continuation of current climatic conditions. These suggests that we should carefully take historically dominant species as the main focus for forest restoration, especially when they are near their natural distribution boundary, because they are probably less capable of successfully adapting to climate change.

Most of the secondary forests in the world display sub-optimal ecological functions^1^,^2^. Therefore, how to rapidly restore the ecological functions of secondary forests is a key issue of forest ecological management^3^,^4^. Planting is known to be an effective measure to accelerate forest succession^5^,^6^ and the dominant species in historical forest communities are usually chosen as the main species for afforestation^7^,^8^. However, forest restoration to the original pre-overexploiting conditions, especially with respect to species composition, can take many years to achieve. Some research has shown that secondary forests can rapidly attain many aspects of structure, environment and diversity of old-growth forests. However, plant species composition would take much longer to fully recover^9^. Some have posited that the Atlantic Rain forest would take 100–300 years just to reach the animal-dispersed species and non-pioneer species levels, while it would take 400 years to reach the endemic levels which exist in mature forests^10^. In the long pathway to restoring secondary forests, many challenges arise due changing environments^11^,^12^, and, in particular, climate change^13^,^14^. The structure of species composition in historical climax communities would likely be altered under climate change^15^,^16^,^17^,^18^.

Forest restoration is not only affacted by changing environments, but also anthropogenic disturbance. In generally, local economies in forest regions are often dependent upon forestry production^19^,^20^, especially timber. Harvesting, the important ecological supply function of forest, would probably hinder forest restoration, especially when it is out of natural bearing capacity^21^. Therefore the balance between ecologcial restoration and ecological supply functions is important for sustainable forest management^22^,^23^. The information of how the dynamics of forests vary under the interaction between ecologcial restoration and ecological supply functions would help forestry policy development^24^,^25^,^26^.

In light of the above, the need to understand and predict the long-term dynamics and development of secondary forests is urgent. Gap models and landscape models are powerful tools which are used to simulate succession of secondary forests under climate change and anthropogenic disturbances. Most research, utilizing models as research tools, has focused on such topics as natural succession of secondary forests^27^,^28^,^29^; effects of planting^5^,^30^,^31^ or harvesting^32^,^33^,^34^; or the combinational effects of planting and harvesting^35^ on secondary forest dynamics without considering climate change; or only addressed climate change without considering planting/harvesting^28^. Relatively little research has addressed combinational effects of planting and harvesting on secondary forest dynamics under climate change. It is a complex question, given that even the effects of harvesting alone may vary considerably^36^. Bu, *et al.*^20^ discussed the tradeoffs between harvesting and planting strategies under possible warming climates in the Khingan Mountains of northestern China. However, the different interaction between these strategies on different scales, the overall forest scale and individual species scale, still remains unaddressed.

The forests in northeastern China are an important component of the world’s temperate forests. As in other temperate forest regions in the world, these forests are experiencing a rapidly changing climate. Although, according to the report of the IPCC 2007, the warming record in mid- and high- attitudes is greater than in the tropics, the precise trajectory of climate change is uncertain. The restoration strategies applied to secondary temperate forests are flexible enough to adapt to this uncertainty. Understanding the individual and interactive effects of different strategies on forest dynamics under climate change would help foresters better understand the interaction between natural and anthropogenic disturbance and forest dynamics and make more adaptive forest management decisions.

In this study, the simulation of the response of secondary forests to anthropogenic disturbance (planting and harvest) under climate change has been carried out in order to examine:

1. Are forest dynamics under continuation of current climate and climate change different? What’s the differences?
2. How do planting efficiencies vary under continuation of current climate and climate change? Do they show same trend? And what the differences?
3. Are there interactive effects of harvest and planting on both the overall forest and individual species?

## Materials and Methods

### The study region

**Figure 1:**
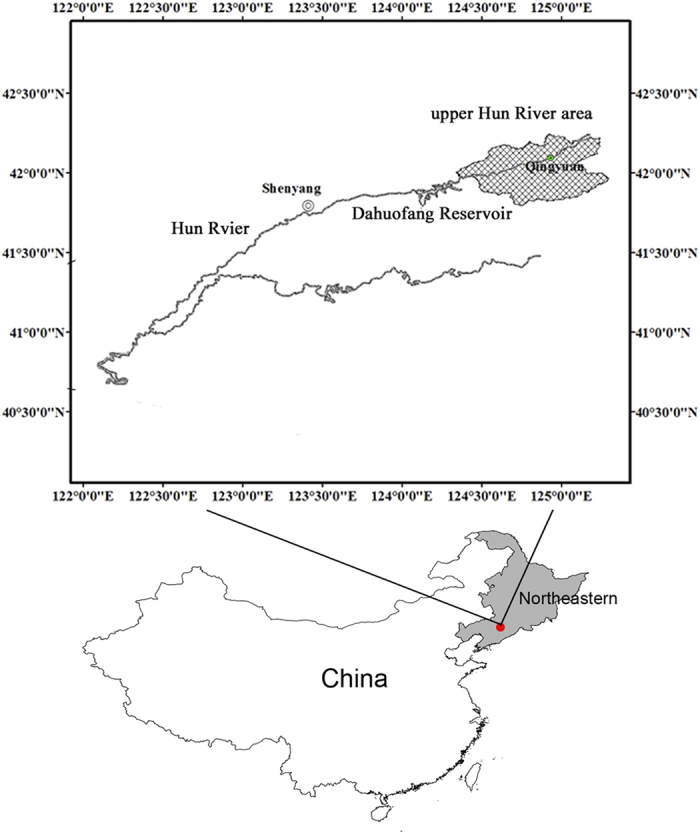
Location of the upper Hun River region (Generated by ArcGIS 9.0 software using Map of China).

This research was conducted in an area encompassing 2.5 × 10^5^ ha in the Changbai Mountain region of Liaoning province in northeastern China (41°47′52″ ~42°28′25″N, 124°20′06″ ~ 125°28′58″E) (Fig. 1). The climate is continental monsoon, with a strong windy spring, a warm and humid summer, and a dry and cold winter^37^. In the past ten years (2000–2010), the mean annual temperature of this region has been 6.7 °C and mean annual precipitation 760 mm, with increasing temperature and relatively stable precipitation levels. The frost-free period lasts 130 days from the start of October to the end of April and the growing season ranges from early April to late September^38^. Because of forest overexploitation, the focal forest area is a typical temperate secondary forest of the region. As opposed to the Korean pine-broad leaved virgin forest in the Changbaishan Nature Reserve, the study area is characterized by mixed forest composed of *Pinus koraiensis, Quercus mongolica, Larix olgensis, Pinus tabulaeformis, Pinus densiflora, Pinus sylvestris var. mongolica, Fraxinus rhynchophylla, Fraxinus chinensis, Juglans mandshurica, Betula platyphylla, Populus davidiana, Acer pictum subsp. mono, Ulmus pumila, Tilia amuresis, Abies nephrolepis, Picea asperata*. Planting *Pinus koraiensis* is the main forest restoration strategy of the study area. Harvest is forbidden for public forest and only open to timer including short-rotation timer, fast-growing timer, general natural timer and general plantation timer.

## Methods

The forest gap model LINKAGES and forest landscape model LANDIS 6.0 were coupled to simulate the natural succession of secondary forests in the upper Hun River region and their response to anthropogenic disturbances (planting and harvesting) under continuation of current climate and climate warming.

### LANDIS 6.0

LANDIS is a spatially explicit, stochastic, raster-based landscape model for simulating forest landscape change at large spatial (10^3^–10^7^ ha) and temporal (10^1^–10^3^ years) scales with flexible resolutions (10–500 m pixel size)^39^. The model includes three general parts: landscape scale ecological processes (seed dispersal, fire and windthrow disturbances, and forest harvesting); environmental data layers (different landtypes represent heterogeneous landscapes); and site or plot scale ecological processes (forest succession dynamics for individual species that were integrated into landscape models depending on the model purpose and technological limitations)^40^. The species establishment coefficients (SEC) are critical parameters of plot scale ecological processes in LANDIS. They encapsulate the effects of environmental factors such as temperature, precipitation and soil, and reflect in a relative sense how different environmental conditions favor a particular species in terms of its establishment. In LANDIS, all sites on the landscape are stratified by different ecoregions (landtype) which may favor certain species over others. In this study SECs were obtained for each species in LINKAGES.

**Figure 2:**
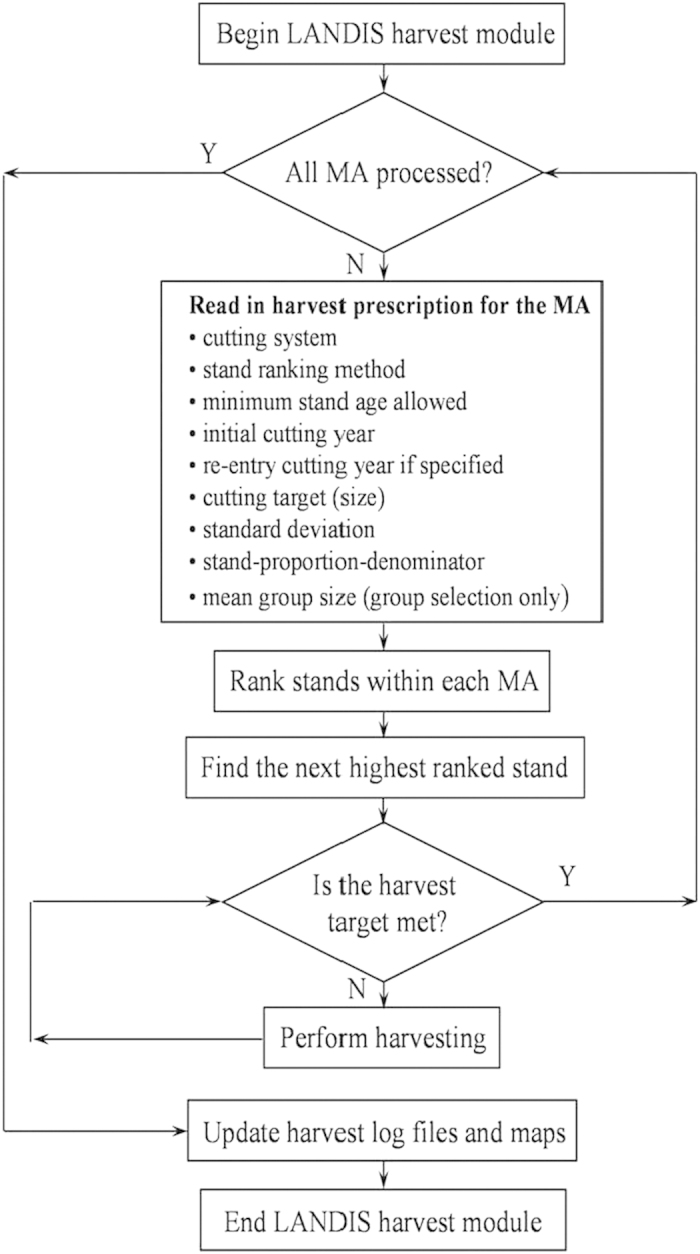
Flow chart of the LANDIS harvest module show harvest actions with one LANDIS iteration.

Planting and harvest are two of landscape scale ecological processes designed for forest dynamic simulation in LANDIS 6.0. They are simulated across two distinct hierarchies of disturbance intensity and the spatial configuration. The spatial configuration of management activity is controlled by the designation of Management Areas (MA) in which distinct management activities and intensities can be simulated on the stands within that MA. The disturbance intensity is controlled by harvest regimes (Fig. 2). There are six harvest regimes in LANDIS 6.0. The “periodic-entry, stand-filling” was chosen in this study. Stand-filling harvest regimes are applied to every site in the stand and do not cross stand boundaries. Stands are prioritized for harvest according to one of four user-specified ranking algorithms. In this study stand age-oldest stands in a management area are harvested first. Stand age is computed as the mean of the oldest cohort on each site within the stand. The harvest mask specifies by species and age class which cohorts will be removed when a harvest regime is applied, and it specifies which species, if any, will be planted. (http://web.missouri.edu/~umcsnrlandis/umcsnrlandispro/landis6.0pro.htm).

**Figure 3:**
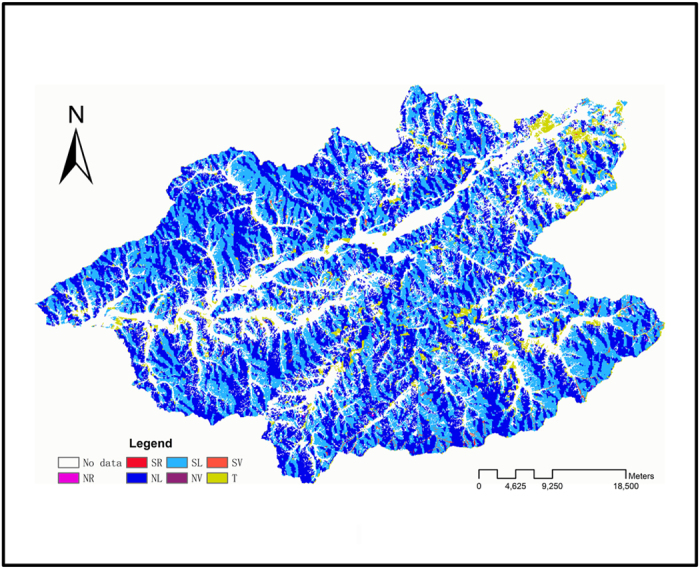
Land types of the upper Hun River region (Generated by ArcGIS 9.0, www.Esri.com).

The data depicting initial forest status in this research was obtained from the 2006 forest inventory of the Qingyuan County Forestry Bureau. Data included forest composition, species distribution, age structure, slope, slope position, and soil type. The landtype in this research was divided into seven components according to slope position and aspect :NorthRidge (NR), North Slope (NL), North Slope of valley (NV), South Ridge (SR), South Slope(SL), South Slope of valley (SV) and terrace (T) (Fig. 3). All model maps in this research were at a resolution of 60 m × 60 m, which yielded 1320 rows × 836 columns. Other parameters for species such as age of maturity, shade tolerance, maximum seeding distance, and so on were derived from the literature on species of this region^41^ and consultation with local experts (Table 1).

Forty-two scenarios were simulated in this research, including natural succession process without anthropogenic disturbances; five levels of planting intensity; three levels of selective harvest intensity; and fifteen different combinations of the five planting intensity levels and three selective harvest intensity levels under current climate and climate change trajectories (Table 2). In the planting scenarios, *P. koraiensis* was planted under broadleaved trees that were greater than 9 years old, since seedlings of *P. koraiensis* require some degree of shade^37^,^42^. In the harvesting scenarios, the harvest standard was from National Forest Resources Continuous Inventory Technique Formula of China (Table 3). According to planting and harvesting options, the study area was divided into 10 management areas (Table 4, Appendix 1). All scenarios were simulated up to 300 years to examine how the forest succession dynamics affected by different intensities of anthropogenic activities (planting and harvesting) under current climate and climate warming over the long run.

### LINKAGES

LINKAGES is a derivative of the JABOWA/FORET class of gap models. It simulates long term dynamics and structure of forest ecosystems at spot scale, especially the physiological response of individual species to environmental change, such as climate change (12-month mean temperature and precipitation) and soil variation (e.g., soil water capacity, total carbon, total nitrogen and wilt point)^43^,^44^.The difference between the SEC for species reflected the different responses of species to climate change. One hundred replications were simulated for each of species. Then SEC of each species equals the successful establishments divided by 100^27^,^45^. (Appendix 2).

The predicted climate used in this research was obtained using the second version of the Canadian Global Coupled Model – IPCC B2 (CGCM2 – IPCC B2). We acquired the climate data at point 127.5°E 43°N which was closest to our research region for forest gap model simulation. The average annual temperature increase prejected at this point over the next 100 years (1990 to 2090) was 4.6 °C and the precipitation variation was < 0.1%.

### statistical methods

To examine the effects of different planting intensities on *P. koraiensis* coverage under current climate continuation and climate change, we calculated how many cells (i.e. areal units) of *P. koraiensis* at year 300 benefited from *P. koraiensis* planting under different planting scenarios. The formula for this is: 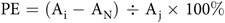
where PE is planting efficiency; A_i_ is the area (cell) of *P.koraiensis* coverage at year 300 under different planting intensity scenarios; A_N_ is the area (cell) of *P.koraiensis* coverage at year 300 in the natural succession scenario without any planting and harvesting; and A_j_ is the overall planting area (cell) in different planting scenarios^24^.

We analyzed the interaction between planting and harvesting on forest composition, and harvest strategies effects on individual species utilizing multivariate analysis of variance (MNOVA) in SPSS 18.0.

## Results

### The response of forestdevelopment to climate change

**Figure 4:**
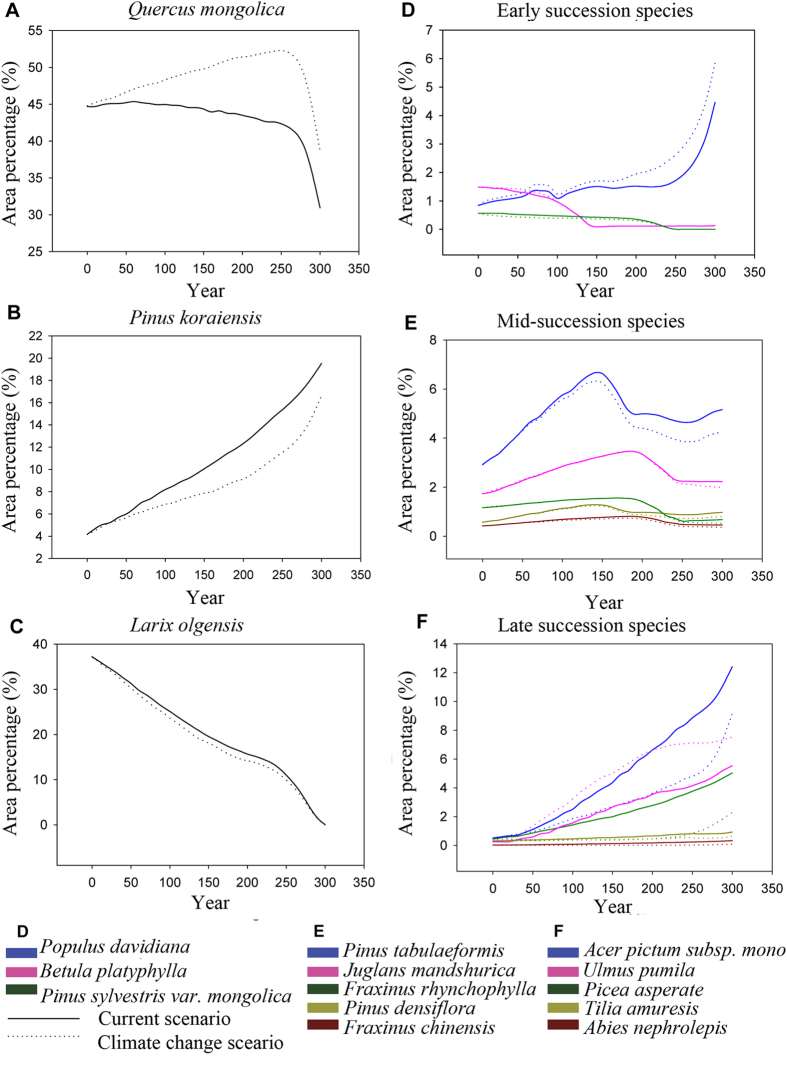
Natural succession dynamics of species in the upper Hun River region under both continuation of current climate and climate warming scenarios.

All species showed different dynamics under the climate change scenario in contrast to the current climate scenario (Fig. 4). The area percentages of *Q. mongolica, P. tabulaeformis* and *U. pumila* were promoted under climate change, while other species were suppressed. While the area percentage of *Q. mongolica* showed a decreasing trend under the current climate scenario, the reverse was true under the climate change scenario. *Q. mongolica* was the dominant species of the study area (occupying 44.7%) in the initial state. At year 300, *Q. mongolica* occupied 30.94% under the current climate scenario and 38.74% under the climate change scenario. It still remained the dominant species in the study area, despite the lower area percentage at year 300. In contrast, the other major species, *P. koraiensis*, displayed increasing area percentage trends, from 4.16% in the initial state to 19.53% and 16.63% under current and future climate scenarios, respectively. The secondary forest recovered very slowly toward the original broad-leaved Korean pine forest.

### The effects of planting strategies on forest dynamics

**Figure 5:**
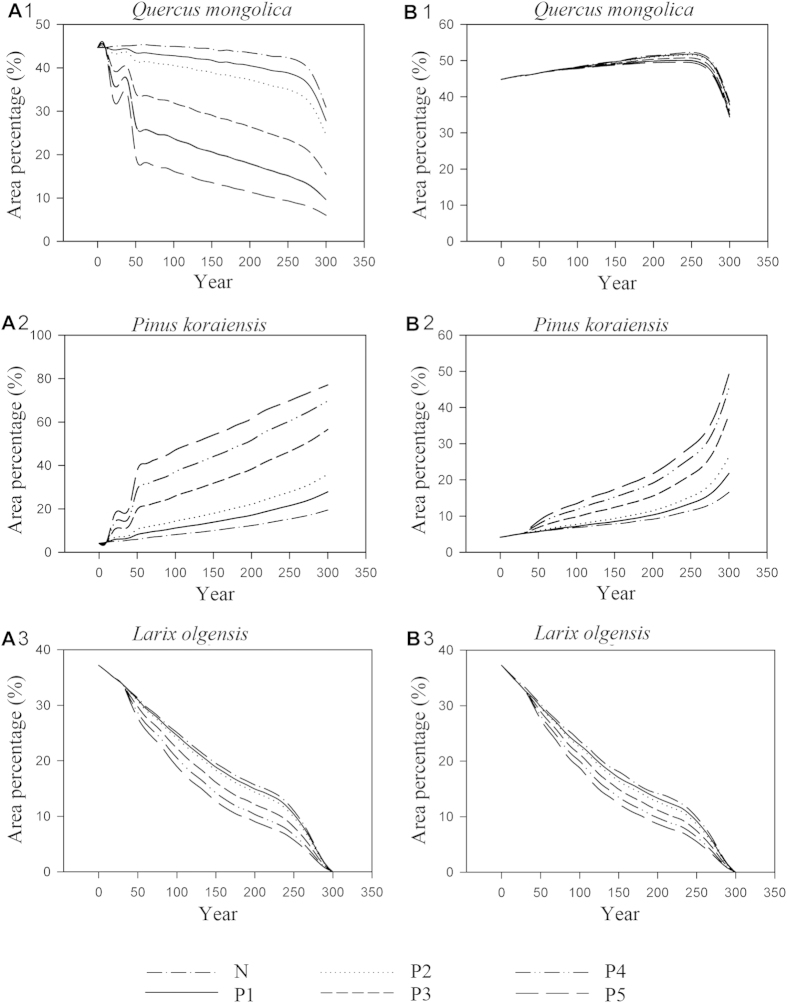
Responses of main species in the upper Hun River region to planting strategies under both continuation of current climate and climate warming scenarios.

**Figure 6:**
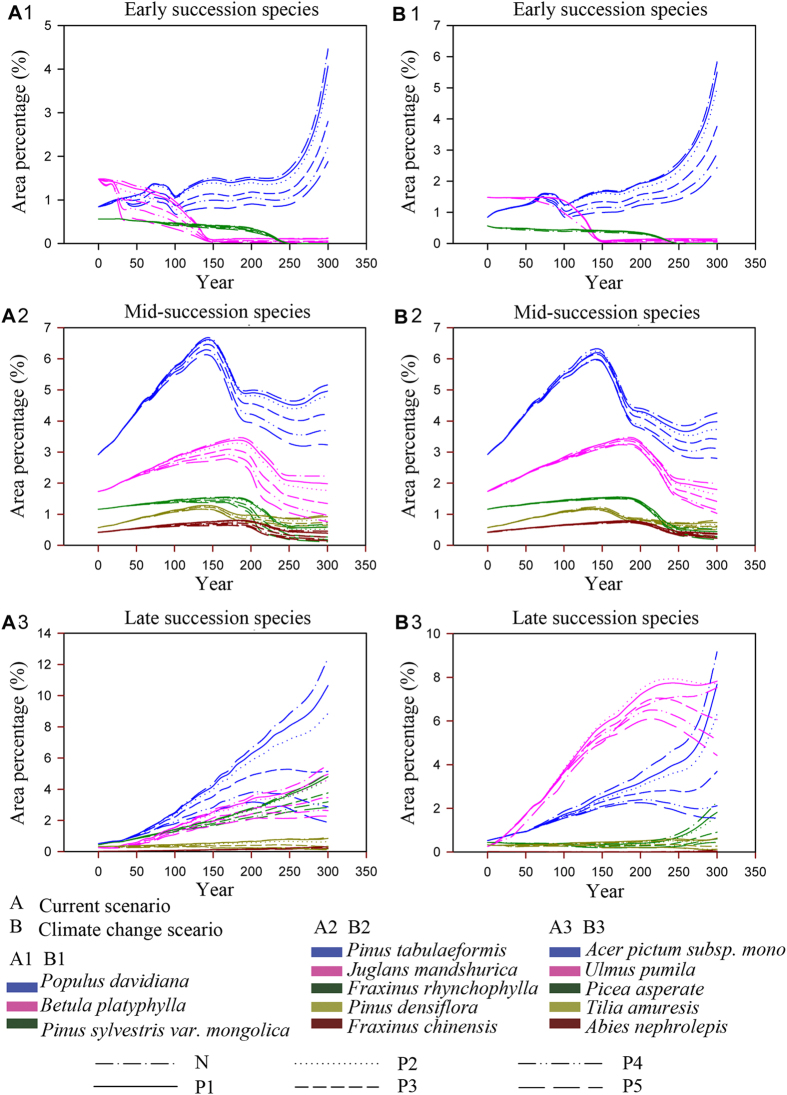
Responses of other species in the upper Hun River region to planting strategies under both continuation of current climate and climate warming scenarios.

Under both climate scenarios, area percentages of *P. koraiensis* were enhanced by planting strategies while other species were suppressed to varying degrees (Fig. 5). Although area percentages of *P. koraiensis* in different planting strategies under the climate change scenario were promoted, the increasing degrees were much lower than under the current climate scenario (Fig. 5). While *Q. mongolica* suffered the greatest loss of living space most under the current climate scenario, it was affected much less under the climate change scenario. Even in the most intensive planting strategy scenario, this species lost less than 10% of the area lost without any restoration effort. Area percentages of most species such as *B. platyphylla, P. sylvestris var. mongolica, P. tabulaeformis, J. mandshurica, F. rhynchophylla, P. densiflora* and *F. chinensis* were more suppressed under the current climate than the climate change scenario (Fig. 6).

In year 300 *Q. mongolica* and *P. koraiensis* were the most important species occupying most of the space in the study area. Under the current climate scenario the area percentages of these two species are nearly the same in the planting strategy P1; while under climate change scenario the area percentage of *P. koraiensis* is slightly higher than that for *Q. mongolica*, even for planting strategy P3 (Fig. 5). For all planting strategies, the area percentages of *P. koraiensis* under the current climate scenario were always higher than those under the climate change scenario (Fig. 5). This was mirrored by the fact that the planting efficiency of *P. koraiensis* was always higher under the current climate scenario (Table 5), although the trends (planting efficiencies decreased with the increasing planting intensity) were same.

### The effects of combination strategies on forest dynamics

There were no significant interaction effects between planting and harvest strategies on species dynamics at the individual species level (data is not shown), but there were significant effects on the overall forest (Table 6). Under the current climate scenario, in the combination strategies, the planting strategies had significant effects on most species, with the exception of *L. olgensis* and *P. sylvestris var. mongolica;* while harvest strategies had no significant effects on most species, with the exceptions being *Q. mongolica, L. olgensis, P. davidiana, B. platyphylla* and *U. pumila*. Under the climate change scenario, planting strategies also had significant effects on most species other than *L. olgensis, P. sylvestris var. mongolica* and *F. rhynchophylla*; while harvest strategies had significant effects on *Q. mongolica, L. olgensis, P. davidiana, B. platyphylla, P. tabulaeformis, P. asperata* and *T. amuresis* (Table 7).

**Figure 7:**
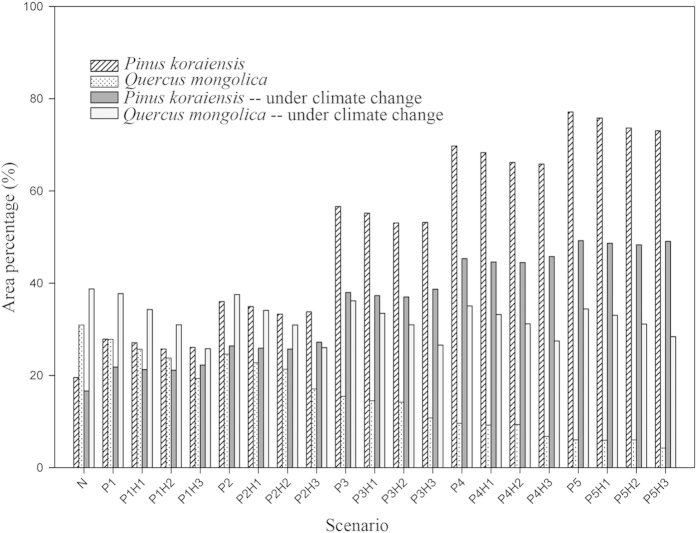
Responses of *Pinus koraiensis* and *Quercus mongolica* to combination strategies under both continuation of current climate and climate warming scenarios.

Area percentages of *Q. mongolica* were suppressed in all combination strategies of planting and harvesting, while area percentages of *P. koraiensis* were promoted (Fig. 7). Under the current climate scenario, the area percentage of *Q. mongolica* was nearly equal to that of *P. koraiensis* in planting strategy P1 and was exceeded by *P. koraiensis* in P1H1. Under the climate change scenario, the area percentage of *P. koraiensis* in planting strategy P2 remained lower than that of *Q. mongolica*, but in combination strategy P2H3, it exceeded that of *Q. mongolica*.

## Discussion

### Biogeographic effects on the response of forest succession to climate warming

Climate change will inevitably have an impact on forest succession dynamics due to the very close relationship between forest structure/composition and climate^46^,^47^,^48^. Although forest succession rates and pathways would likely be affected by climate change for species-specific responses to environmental variability^46^,^49^,^50^,^51^, such responses would obviously vary. A few researchers have compared rates or pathways of secondary succession across broad climate gradients. Some demonstrated that the succession of forests in more mesic regions is more rapid than in more arid regions^52^,^53^,^54^. Prach, *et al.*^55^ found that in the Czech Republic, mean annual change in dominant species cover during the first 12 years of succession increased dramatically with decreasing precipitation and increasing temperature. In our research, the area of historically dominant species increased more slowly under projected climate change (increasing temperature and non-significant change in precipitation) than it did under continuation of current climate. Thus we can conclude that increasing precipitation or increasing temperature is not necessarily good for a species. We infer that the biogeography factor probably counts a lot. If the species is at the edge of its distribution area, it would be sensitive to climate variations (temperatrue and precipitation), and thus the succession trajectories in these areas would be substantially affected under climate change. On the other hand, in the central regions of a species’ distribution, the varied climate is always within the scope of species’ adaptive capacities to climate change, so forest succession trajectories are less affected and would likely be in the range of the natural variability domain. For example, in the Northern Hemisphere, when temperature is the critical factor for vegetation and the dominant species is distributed at the northern edge or middle of its natural range, increasing temperature would promote growth of the original dominant species and consequently promote forest succession^56^,^57^. If the dominant species is at the southern edge of its natural range, the species would probably shift northward and the pathway of forest succession in the original area would change^58^,^59^. In our case, the historically dominant species *P. koraiensis* and the currently dominant species *Q. mongolica* show different responses to climate change. The latter seems more adaptive to a changing climate than does *P. koraiensis*. This result is consistent with findings of previous research in the Changbai Nature Reserve and Qingyuan Forest Ecosystem Experiment Station, which is located in our study area^27^,^60^. Oak is considered to be a drought- and heat-tolerant species, so it would adapt to the warmer and drier climate in the future, while *P. koraiensis* favors a cooler climate. Our study area is located in eastern Liaoning province, which is close to the southern edge of *P. koraiensis*’ distribution and in the central region of *Q. mongolica*’s distribution. In this light, *P. koraiensis* would be more sensitive to climate change in our study area, while *Q. mongolica* would better adapt to a change in climate. Thus forest succession toward a historical climax community would probably be delayed or even deviate from its historical trajectory.

### Planting effeciency is affected by the change of species’ fundamental and realized niches

In the climate warming scenario, the trend for planting efficiency of *P. koraiensis* is lower (Table 5). Our results suggest that the species chosen for ecological restoration according to the historical climax forest community will probably not successfully adapt to the changed environment. Numerous research has shown that species adapted to cooler climate are not adapted to the warmer climate. Also, Species adaptation to environmental change is affected not only by inherent adaptive capacity, but also by many other factors, such as competition, extent of species dispersal, anthropogenic disturbance and other interactions among organisms. Environmental changes may induce changes of fundamental niche and the realized niche of species and species assemblages may not shift in unison. In our study, given the difference in *P. koraiensis’* planting effeciency between different climate scenarios, we infer that the fundamental and realized niches of *P. koraiensis* change under the climate change scenario. There are probably two reasons for this: (1) The adaptive capacity changes because of the changed environment. The southern boundary of *P. koraiensis’* original distribution is 40°45′N and the species favors a cooler climate^61^,^62^. It is projected to move northward in the Northern Hemisphere under climate change^62^,^63^. (2) Interspecific competition changes. Under the warmer climate scenario, the spread of *P. koraiensis* is suppressed, while that of *Q. Mongolica* is enhanced (Fig. 4). This suggests that the relative competitiveness of *P. koraiensis* decreases^61^,^64^. The change of the relative competitiveness between *P. koraiensis* and *Q. mongolic* indicates that the niches of species assemblage do not shift in unison and the change in their realized niches is induced by both the change of basic niches and the change in competition.

Although the species assemblage in our study area evolves slowly towards the historical climax community, there are still two key points that should be noted. First, at the present stage the realized niche of *P. koraiensis* is narrow. Due to anthropogenic disturbance, there is a lack of seed source and thus the spread of *P. koraiensis* is limited. Secondly, planting is an effective measure for assisting focal species in area expansion, which is good for the spread of species’ realized niches. However, if the fundamental niche shrinks due to environmental change, the expansion of realized niches via expanding the seed source artificially (e.g., via planting) would likely be counter to the natural development trend and induce low efficiency. In this light, research on shifts in species’ fundamental niches under environmental change is essential for ecological restoration. Expanding realized niches of dominant species of historical climax communities without sound reasons would probably work against natural successional processes and the principle of economy and high efficiency in forest management.

### The response of forest dynamics to timber harvesting

Harvesting is one of the key components of sustainable forest management.It is both one of methods of providing ecological services from forests but can also be the cause of forest degradation, which depends on the balance between harvest intensity and forest ecological capacity. Most research has focused on the response of community dynamics to different harvesting intensities and explored which level of harvesting would best contribute to sustainable forest management. Some studies have investigated forest responses to different harvesting intensities under different climate scenarios^20^. In our research, the response of forest dynamics to harvesting and combined planting-harvesting policies under continuation of current climate and warming climate scenarios are discussed.

1. *Organizational scale effect*. In our study, the combination of planting and harvesting had significant effects on the overall forest but not on the individual species (Table 6). Scale effects may be temporal, spatial, or organizational in nature. At present, the former two have received much more attention than the latter^65^. Organizational scale effects are always context dependent. Peckarsky, *et al.*^66^ found that large scale patterns of distribution and abundance of organisms sometimes deviate from those expected from patterns of individual behavior and hypothesized that such discrepancies are often due to multiple confounding factors, including abiotic disturbances, that have effects on populations and communities at larger scales. Herben and Goldberg^67^ showed that changes in community-wide trait means can have large effects on diversity for a given degree of dispersion even if the relationship between dispersion and diversity doesn’t change, because the effect of changes in value of a given trait depends on the values of other traits, both for an individual species and for other species in the community. In our research, the different responses of the overall forest and individual species to the combination policy of planting and harvesting are also context dependent. They depend on the intensity of policy elements, forest composition, forest structure, and so on^36^,^65^.
2. Warming climate affects species adaptation, which induces the different responses of species to harvesting^68^. Some species display no significant responses to harvesting under the continuation of current climate scenario, but are more sensitive to harvesting under warming climate conditions (Table 8). These include *B. platyphylla, Q. mongolica, T. amuresis* and *P. asperata*. It is evident here that, under the warming climate scenario, the trends in area variation of these species are suppressed, while that of *Q. mongolica*is enhanced. In this light, the reason for the change of species sensitivity to harvesting likely arises from the relationship between the extent of the area change for a species and the unchanging harvest intensity.

## Conclusion

Species with different geographical distributions are differentially affected by climate change, as are species assemblages of the same flora in different geographic localities. These effects are reflected in fundamental and realized niches of historically dominant species and the resulting changes in competition triggered by a changing climate. As a result, the direction and path of forest succession and forest restoration would probably deviate from the historical track if historically dominant species are close to or at the boundary of their distributions, since they are more sensitive to environmental changes. In this research, the historically dominant species *P. koraiensis*is close to its southern distributional boundary and displays decreasing competitiveness, as reflected to some degree in decreasing trends in area expansion and planting efficiency. This leads to a caveat: we should pay attention to geographical distributions and shifts in the fundamental niches of species, when we choose a species for forest restoration, especially under climate change.

The balance between forest restoration and harvesting is crucial to ecological protection and human well-being. Given that the interaction between planting and harvesting of forests has organizational scale effects, exploring the impacts of harvesting on forest restoration dynamics at multi-scale levels would benefit the sustainable development of forest ecosystems at a time when the future pathway of the planet’s climate has become an urgent concern.

## Additional Information

**How to cite this article**: Yao, J. *et al.* The long-term effects of planting and harvesting on secondary forest dynamics under climate change in northeastern China. *Sci. Rep.* **6**, 18490; doi: 10.1038/srep18490 (2016).

## Supplementary Material

Supplementary Information

## Figures and Tables

**Table 1:** Species’ key attributes for secondary forests in the upstream Hun River in northeastern China.

Species	LONG	MTR	ST	FT	ED	MD	VP	MVP
*Quercus mongolica*	350	40	3	5	20	200	0.9	60
*Pinus koraiensis*	400	40	5	1	50	200	0	0
*Populus davidiana*	100	8	1	2	−1	−1	1	10
*Larix olgensis*	300	30	1	5	100	400	0	0
*Pinus densiflora*	200	30	2	1	100	500	0	0
*Acer pictum subsp. mono*	250	10	4	2	120	350	0.3	50
*Juglans mandshurica*	250	15	3	4	50	150	0.9	60
*Fraxinus chinensis*	250	30	3	3	50	150	0.3	80
*Fraxinus rhynchophylla*	250	30	3	3	50	150	0.3	80
*Pinus tabulaeformis*	200	30	2	1	100	500	0	0
*Pinus sylvestris var. mongolica*	250	40	2	2	30	100	0	0
*Picea asperata*	300	30	5	3	80	150	0	0
*Abies nephrolepis*	250	40	5	3	80	150	0	0
*Betula platyphylla*	150	15	1	1	200	4000	0.8	50
*Ulmus pumila*	250	10	2	4	300	1000	0.3	60
*Tilia amuresis*	300	30	4	4	50	100	0.9	30

Long—longevity (years); MTR—age of maturity (years); ST-shade tolerance class; FT—fire tolerance class; ED—effective seeding distance (m); MD—maximum seeding distance (m); VP—vegetative reproduction probability; MVP—minimum age of vegetative reproduction (years).

**Table 2:** The management strategies (planting and harvesting) scenarios simulated by LANDIS 6.0.

Scenarios	PI	SHG	SHS
N	—	—	—
P1	5%	—	—
P2	10%	—	—
P3	30%	—	—
P4	50%	—	—
P5	70%	—	—
P1H1	5%	10%	30%
P1H2	5%	30%	50%
P1H3	5%	50%	70%
P2H1	10%	10%	30%
P2H2	10%	30%	50%
P2H3	10%	50%	70%
P3H1	30%	10%	30%
P3H2	30%	30%	50%
P3H3	30%	50%	70%
P4H1	50%	10%	30%
P4H2	50%	30%	50%
P4H3	50%	50%	70%
P5H1	70%	10%	30%
P5H2	70%	30%	50%
P5H3	70%	50%	70%

PI—Planting intensity of *P. koreaiensis.*

SHG—Selective Harvest intensity for general timber forest.

SHS—Selective Harvest intensity for short-rotation forest and fast-growing forest.

Note: 1. *P. koraiensis* was planted under broadleaved trees that were >9 years old.

2. Those 21 scenarios are simulated both under current climate and climate warming.

**Table 3:** Harvesting standars of species derivated from National Forest Resources Continuous Inventory Technique Formula (China).

Species	GN	GP	FG	SR
*Quercus mongolica*	>80	>50	—	—
*Pinus koraiensis*	>120	>80	—	>40
*Populus davidiana*	>20	>20	>20	>10
*Larix olgensis*	>100	>40	>20	>20
*Pinus densiflora*	>100	>40	—	—
*Acer pictum subsp. mono*	>80	>50	—	>30
*Juglans mandshurica*	>80	>50	—	>20
*Fraxinus chinensis*	>80	>50	—	>20
*Fraxinus rhynchophylla*	>80	>50	—	>20
*Pinus tabulaeformis*	—	—	>40	>60
*Pinus sylvestris var. mongolica*	>100	>40	—	>20
*Picea asperata*	>120	>80	—	—
*Abies nephrolepis*	>100	>40	—	—
*Betula platyphylla*	>60	>40	>20	>10
*Ulmus pumila*	>60	>40	—	—
*Tilia amuresis*	>80	>50	—	—

GN—general natural timber; GP—general plantation timber; FG—fast-growing timber; SR—short-rotation timber.

**Table 4:** The 10 management areas of this research in LANDIS 6.0.

Management area	Forest type	Forest detail information		Harvest
	BT > 9 years old	All trees except BT > 9 years old		
MA1	SR	√		Y
MA2	SR		√	Y
MA3	FG	√		Y
MA4	FG		√	Y
MA5	PF	√		N
MA6	PF		√	N
MA7	GN	√		Y
MA8	GN		√	Y
MA9	GP	√		Y
MA10	GP		√	Y

SR—short-rotation timber forest; FG—fast-growing timber forest; PF—public forest; GN—general natural forest; GP—general plantation forest; BT—broadleaf tree.

**Table 5:** Panting efficiency of ***P. koraiensis*** in different planting intensity under current climate and climate warming at year 300.

Scenarios	Planting Intensity(%)	Area Percentage at Year 300 (%)		Area Percentage Increase (%)		Planting Efficiency	
		CC	CW	CC	CW	CC	CW
P1	5	27.87	21.81	8.34	5.18	3.6	2.23
P2	10	35.99	26.40	16.46	9.77	3.54	2.11
P3	30	56.63	37.99	37.10	21.36	2.67	1.53
P4	50	69.76	45.32	50.23	28.69	2.17	1.24
P5	70	77.14	49.23	57.61	32.60	1.77	1.00

CC—Current Climate; CW—Climate Warming.

**Table 6:** The interaction between planting and harvesting on forest composition.

Effect	Pliiai’s		Wilks’ Lambda		Hotlling’s Trace		Roy’s Largest Root	
	CC	CW	CC	CW	CC	CW	CC	CW
Value	0.164	0.775	0.836	0.419	0.196	0.985	0.196	0.448
Sig.	<0.001	<0.001	<0.001	<0.001	<0.001	<0.001	<0.001	<0.001

CC—Current Climate; CW—Climate Warming.

**Table 7:** Contrast estimate of effects of different harvest intensity on area percentage of species for individual species.

Dependent variables	Level 2 vs. level 1				Level 3 vs. level 1			
	Current climate		Climate change		Current climate		Climate change	
	Contrast Estimate	Sig.	Contrast Estimate	Sig.	Contrast Estimate	Sig.	Contrast Estimate	Sig.
*Pinus koraiensis*	−0.627	0.690	−0.053	0.957	−0.648	0.680	−0.053	0.957
*Pinus tabulaeformis*	0.065	0.545	−0.100	0.258	0.148	0.167	−0.100	0.258
*Pinus densiflora*	0.150	0.467	−0.014	0.500	0.049	0.020	−0.014	0.500
*Pinus sylvestris var. mongolica*	−0.020	0.366	−0.016	0.447	−0.017	0.442	−0.016	0.447
*Larix olgensis*	−2.127	0.087	−1.952	0.116	−4.252	0.001	−1.952	0.116
*Picea asperata*	0.008	0.949	0.086	0.001	0.047	0.711	0.086	0.001
*Abies nephrolepis*	6.452E-5	0.993	−0.001	0.160	−0.009	0.219	−0.001	0.160
*Populus davidiana*	1.548	<0.001	1.971	<0.001	3.847	<0.001	1.971	<0.001
*Betula platyphylla*	0.054	0.220	0.101	0.081	0.145	0.001	0.101	0.081
*Ulmus pumila*	0.493	0.018	0.225	0.531	0.961	<0.001	0.225	0.531
*Fraxinus chinensis*	0.003	0.819	−0.018	0.201	0.014	0.358	−0.018	0.201
*Fraxinus rhynchophylla*	−0.003	0.942	−0.026	0.547	0.008	0.857	−0.026	0.547
*Juglans mandshurica*	0.022	0.723	−0.036	0.557	0.086	0.172	−0.036	0.557
*Quercus mongolica*	−0.567	0.555	−1.289	0.001	−3.156	0.001	−1.289	0.001
*Acer pictum subsp. mono*	0.053	0.830	0.031	0.832	0.065	0.792	0.031	0.832
*Tilia amuresis*	0.004	0.771	0.002	0.799	0.028	0.024	0.002	0.799

Level1: selectively harvesting 10% of general timber forest and 30% of other timber forest.

Level2: selectively harvesting 30% of general timber forest and 50% of other timber forest.

Level3: selectively harvesting 50% of general timber forest and 70% of other timber forest.

**Table 8:** Responses of individual species to harvesting under current climate and climate change (warming).

Species	Type III Sum of Squares		F		Sig.	
	CC	CW	CC	CW	CC	CW
*Pinus koraiensis*	0.28	27.929	0.000	0.190	0.990	0.827
*Pinus tabulaeformis*	1.076	3.948	1.225	3.267	0.269	0.039
*Pinus densiflora*	0.071	0.015	2.102	0.241	0.148	0.786
*Pinus sylvestris var. mongolica*	0.006	0.021	0.164	0.301	0.686	0.740
*Larix olgensis*	364.19	1201.998	3.068	5.058	0.081	0.007
*Picea asperata*	0.050	1.611	0.041	15.863	0.840	<0.001
*Abies nephrolepis*	0.002	0.000	0.470	1.582	0.490	0.207
*Populus davidiana*	361.468	1770.470	89.346	132.993	<0.001	<0.001
*Betula platyphylla*	0.652	3.976	4.346	7.767	0.038	<0.001
*Ulmus pumila*	24.840	22.714	7.530	1.144	0.006	0.319
*Fraxinus chinensis*	0.000	0.073	0.008	2.382	0.928	0.094
*Fraxinus rhynchophylla*	0.002	0.112	0.011	0.376	0.918	0.687
*Juglans mandshurica*	0.013	0.496	0.043	0.839	0.836	0.433
*Quercus mongolica*	450.840	1741.727	6.242	77.955	0.013	<0.001
*Acer pictum subsp. mono*	0.450	0.240	0.097	0.074	0.755	0.929
*Tilia amuresis*	0.016	0.064	1.314	6.732	0.252	0.001

CC—Current Climate; CW—Climate Change (Warming).
