# Supplementary material for: The long-term effects of planting and harvesting on secondary forest dynamics under climate change in northeastern China
Source: Sci Rep. 2016 Jan 4;6:18490. doi: 10.1038/srep18490 (PMC4698755; doi:10.1038/srep18490)
Supplement: Supplementary Information [file srep18490-s1.pdf]

The long-term effects of anthropogenic disturbances on secondary forest

The long-term effects of planting and harvesting on secondary forest dynamics under climate change in northeastern China

Jing Yao[ab], Xingyuan He[ab*], Hongshi He[a], Wei Chen[a], Limin Dai[a], Bernard J.Lewis[a], LizhongYu[ab]

[a] State Key Laboratory of Forest and Soil Ecology, Institute of Applied Ecology, Chinese Academy of Sciences, Shenyang 110164, China

[b] Qingyuan Forest CERN, Chinese Academy of Sciences, Shenyang 110016, China

** Corresponding author. Tel.: +86 24 83970349; fax: +86 24 83970300*

E-mail addresses: xyhe_1828@163.com; hexy@iae.ac.cn

Appendix 1 The management areas the upper Hun River region in LANDIS (Generated by ArcGIS 9.0, WWW. Esri. com)

Appendix 2 The species establishment coefficients under current climate and climate change

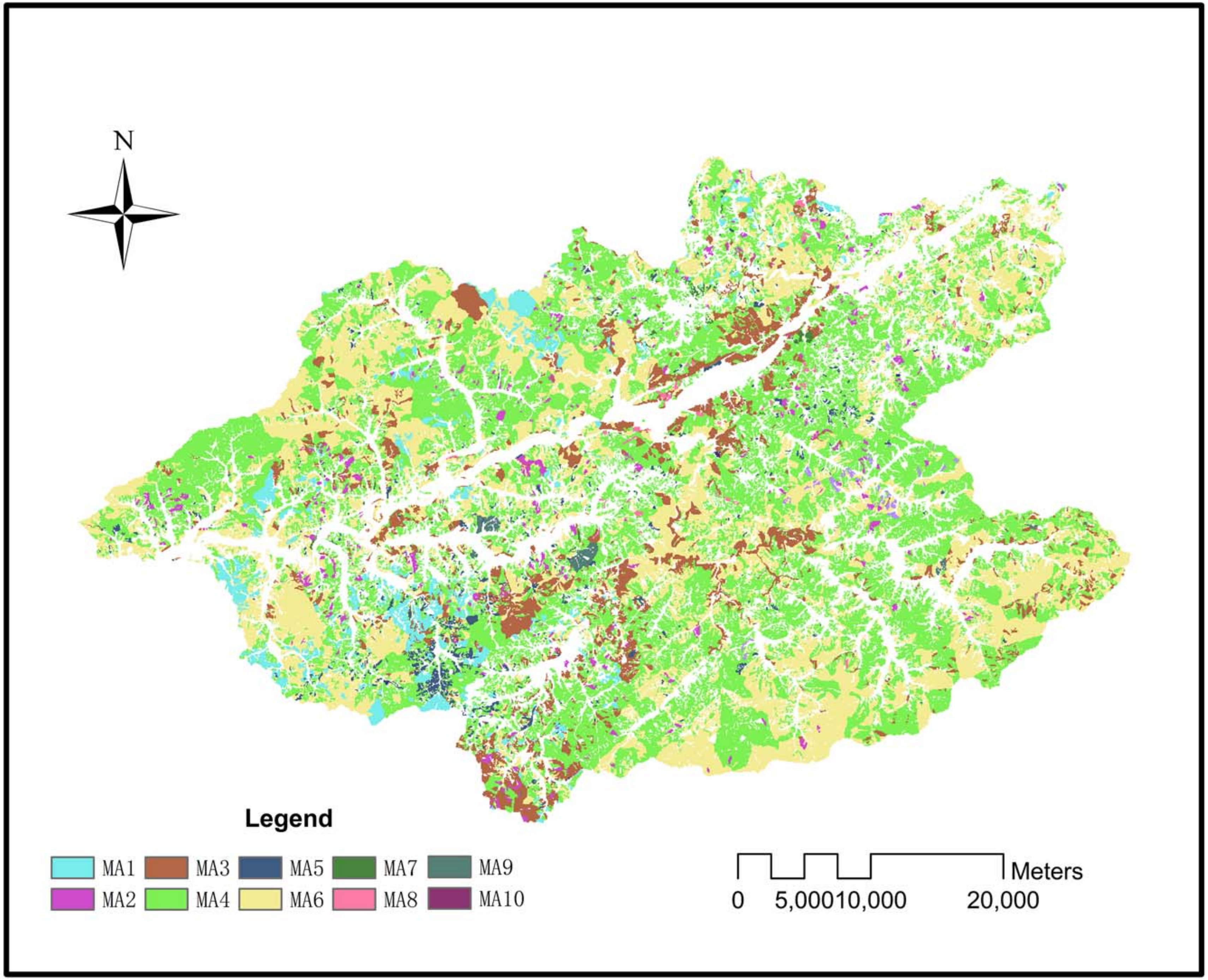
N
Legend
MA1
MA2
MA3
MA4
MA5
MA6
MA7
MA8
MA9
MA10
0
5,000
10,000
20,000
Meters

Appendix 1

Appendix 2

The species establishment coefficients under current climate and climate change

species	SECs under current climate							SECs under climate change						
	NR	SR	NL	SL	NV	SV	T	NR	SR	NL	SL	NV	SV	T
Pinus koraiensis	0.62	0.42	0.70	0.60	0.72	0.75	0.55	0.56	0.35	0.65	0.50	0.67	0.64	0.55
Pinus tabulaeformis	0.00	0.00	0.33	0.22	0.34	0.23	0.24	0.00	0.00	0.40	0.20	0.31	0.25	0.21
Pinus densiflora	0.00	0.00	0.32	0.23	0.31	0.20	0.21	0.00	0.00	0.38	0.25	0.29	0.20	0.18
Pinus sylvestris var. mongolica	0.00	0.00	0.20	0.18	0.00	0.00	0.00	0.00	0.00	0.15	0.11	0.00	0.00	0.00
Larix olgensis	0.00	0.00	0.10	0.08	0.14	0.05	0.00	0.00	0.00	0.11	0.12	0.10	0.04	0.00
Picea asperata	0.00	0.00	0.65	0.55	0.43	0.00	0.11	0.00	0.00	0.55	0.43	0.36	0.00	0.12
Abies nephrolepis	0.00	0.00	0.59	0.52	0.54	0.33	0.20	0.00	0.00	0.18	0.15	0.15	0.10	0.10
Populus davidiana	0.25	0.32	0.12	0.21	0.00	0.00	0.22	0.27	0.35	0.10	0.35	0.00	0.00	0.33
Betula platyphylla	0.10	0.05	0.11	0.19	0.00	0.00	0.22	0.12	0.04	0.09	0.24	0.00	0.00	0.30
Ulmus pumila	0.00	0.00	0.31	0.32	0.33	0.32	0.30	0.00	0.00	0.45	0.44	0.45	0.42	0.00
Fraxinus chinensis	0.00	0.00	0.45	0.43	0.00	0.00	0.40	0.00	0.00	0.57	0.53	0.00	0.00	0.60
Fraxinus rhynchophylla	0.00	0.00	0.41	0.39	0.42	0.41	0.45	0.00	0.00	0.50	0.42	0.52	0.50	0.52
Juglans mandshurica	0.00	0.00	0.43	0.45	0.00	0.53	0.47	0.00	0.00	0.48	0.50	0.00	0.56	0.53
Quercus mongolica	0.55	0.70	0.20	0.44	0.10	0.22	0.60	0.67	0.78	0.45	0.56	0.23	0.40	0.72
Acer pictum subsp. mono	0.00	0.00	0.53	0.33	0.32	0.40	0.31	0.00	0.00	0.60	0.45	0.50	0.61	0.48
Tilia amuresis	0.00	0.00	0.43	0.43	0.45	0.40	0.39	0.00	0.00	0.22	0.25	0.30	0.20	0.31

NR—north ridge, SR—south ridge, NL—north slope, SL—south slope, NV—north slope of valley, SV—south slope of valley, T-- terrace

SECs—species establishment coefficients
